# What Will Happen to Humanity in a Million Years? Gilbert Hottois and the Temporality of Technoscience

**DOI:** 10.1007/s13347-025-00887-4

**Published:** 2025-04-29

**Authors:** Massimiliano Simons

**Affiliations:** Department of Philosophy, Maastricht University, Maastricht, The Netherlands

**Keywords:** Gilbert Hottois, Technoscience, Guidance ethics, Philosophy of time, Practice turn, Empirical turn

## Abstract

This article provides an overview of the philosophy of Gilbert Hottois, who is usually credited with popularizing the concept of technoscience. Hottois starts from a metaphilosophy of language that diagnoses twentieth-century philosophy as fixated on language at the expense of technology. As an alternative, he developed a philosophy of technoscience that reinterprets science as primarily an intervening and technical activity rather than a contemplative and theoretical one. As I will argue, Hottois articulates the nature of this technicity through a philosophy of time, reflecting on the specific temporality of technoscience as distinct from human history. This temporality of technoscience provoked the need for ethical reflection, since technoscience is constantly changing and transforming the world. This led to Hottois’s engagement with bioethics, in which he sought to develop a framework capable of “guiding” technoscience. Aiming to avoid both total symbolic closure and total technical openness, this guidance is concerned with the preservation of diversity, especially the human capacity for ethics, ethicity. This idea of guidance was later taken up by Dutch philosophers such as Hans Achterhuis and Peter-Paul Verbeek, inspiring their empirical turn in the philosophy of technology. What remains missing in this framework, however, is Hottois’s critical analysis of the different temporalities at work in technology and culture.

“If man is to be the measure of techno-science, it is essential that he should not be affected in his essence by what he measures.” (Hottois 1984b, 23)

## Introduction

“What will become of man in a million or ten million years?” (Hottois, 1984a, p. 83) This question is at the heart of the work of the Belgian philosopher Gilbert Hottois. What makes this question central to Hottois’s philosophy is not so much its possible answer, as transhumanists and longtermists seek, but rather the *modality* of the question: What are the implications for philosophy when such a question becomes a meaningful question to ask? For Hottois, it is a meaningful question, but one that is in principle unanswerable, and its significance lies precisely in its unanswerability. It is this radical opacity that shows, for Hottois, that it is fundamentally different from the traditional *historical* questions. Whereas historical questions elicit meaningful responses (where did we come from? where are we going?), this question remains fundamentally open because it is technology that constitutes the condition of possibility for this question. As I will argue in this article, Hottois argues that technology allows us to formulate this question, but also prevents us from answering it, since technology introduces a fundamental temporal opacity and openness. Through technology we experience that the future is fundamentally unpredictable for us. Hence his plea for a reconceptualization of contemporary science as ‘technoscience’ in order to enable us to recognize this specific temporality of technoscience and to find a way to guide it ethically.

The aim of this article is to provide an introduction to Gilbert Hottois’s philosophy of technoscience. Hottois is generally credited with popularizing the concept of technoscience, which has been taken up by authors such as Jean-François Lyotard (1986), Latour (1987), Stiegler (1994), Haraway (1997), Ihde (2009) and Bernadette Bensaude-Vincent (2009). More broadly, Hottois’s philosophy of technoscience can also be seen as an early example of the ‘practice turn’ in philosophy of science (Soler et al., 2014). The late 1970s saw a boom in authors emphasizing the experimental and technological side of science, leading to a number of calls for a reconceptualization of philosophy of science (see Potters & Simons, 2023). We will see that Hottois’s case presents us with a specific version of the practice turn for at least three reasons.

First, Hottois approaches this question through a critical dialogue with a radically different tradition. His work can be seen as developed primarily in response to the Francophone biological philosophy of technology, a tradition associated with Henri Bergson, André Leroi-Gourhan, Gilbert Simondon, and Bernard Stiegler (Loeve et al., 2018; Clarizio, 2021). And although authors in this tradition have recently received much attention (e.g. Bardin, 2015; Schlanger, 2023), Hottois's work has so far been ignored. Indeed, there is no overview of his work in English, although there are small studies in French (Dumoncel, 1985; Goffi, 1988) and Dutch (Achterhuis, 1998; De Dijn, 2000).

But there is a second reason why his practical turn differs from what is happening in Anglophone philosophy of science: his turn to technology is also a turn to ethics, in particular to a framework for ‘guiding’ technological developments. This has to do with a second strand of Hottois’s legacy, which is worth mentioning but has remained even more hidden. Hottois’s work was influential in the way the ‘empirical turn’ (Achterhuis, 2001) in Dutch philosophy of technology took shape, in particular the so-called mediation theory (Verbeek 2005) and the ethical frameworks that followed from it (Verbeek, 2008, 2011). Both Achterhuis and Verbeek explicitly borrow this concept of ‘guidance’ from Hottois. However, as I will return to in the conclusion, Hottois’s legacy to this tradition, while profound, is also selective, especially with regard to the temporality of technoscience. Although the Dutch philosophers of the empirical turn take up Hottois’s message that both technology and morality are in constant flux, they do not take up his other crucial and related insight, namely that technology and symbols have fundamentally different kinds of temporalities, raising questions about potential incongruities between these temporalities. This insight could potentially be the basis for another critical project that analyzes and evaluates technologies and cultural practices through their different temporalities.

This selective reception also points to a third reason, related to an ambiguity within the ‘practice turn’. In fact, the latter can mean two different things. Today, it is most often understood as a methodological claim: philosophy of science should study science through the lens of concrete scientific practices and case studies. This is often combined with a second, ontological claim about the nature of science: science is not a set of theories, but a set of (technological) practices. While these often go hand in hand, this is not the case for Hottois. He follows the latter, but not the former. Instead, Hottois arrives at the ontological claim in a different way, namely by drawing mainly on science fiction (Hottois, 2000, 2022) and by exploring a series of speculative questions and scenarios. Thus, while Hottois and the Dutch philosophers agree on the ontological claim, they differ in their methodology. As we shall see, it is this speculative method that allows Hottois to make his claims about incongruent temporalities, while the Dutch philosophers borrow only what they can use for their methodological program.

The general claims of this article are as follows. First, I will argue that the work of Gilbert Hottois can be divided into three distinct periods, which will also give the article its structure. A first period in the 1970s, in which Hottois focuses on the philosophy of language. A second period in the 1980s, in which Hottois turns to questions of technology and develops a philosophy of technoscience. A final period, beginning in the 1990s, in which Hottois’s philosophy shifts towards bioethics. My second claim is that Hottois’s philosophy of technoscience should be seen as a philosophy of time. In other words, Hottois’s concept of technoscience aims to articulate the specific duration of science and technology, which differs from the duration of human symbols and meanings. Finally, I will argue that this temporality of technoscience leads him to reconceptualize the task of philosophy of science as fundamentally ethical. If science is not about representing but about operating in the world, then the main philosophical question is not the epistemic assessment of the relation of representation, but the practical question of the ethical implications of these operations. This reconceptualization underlies Hottois’s later work in bioethics.

## A Metaphilosophy of Language

Gilbert Hottois (1946–2019) was a true philosopher of Brussels: he was born there, he studied and worked there, and died there. He studied Romance philology and then philosophy at the Francophone Université Libre de Bruxelles (ULB). This led to his first book on Ludwig Wittgenstein’s philosophy of language (Hottois 1976). In that year, he began his doctoral research, under the supervision of Jean Paumen, which led to a dissertation on “The Inflation of Language in Contemporary Philosophy: Causes, Forms, and Limits”, which was published in an abridged version two years later (Hottois, 1979). In the same year; he was offered a permanent position at the ULB, where he would remain for the rest of his life.

Hottois’s dissertation continued his fascination with the philosophy of language. Although it remains a central reference in his own work, his next two books are in fact more accessible summaries of the (long) dissertation. While *Pour une métaphilosophie du langage* (1981) is a summary of the first part of the dissertation, on language, *Le signe et la technique* (1984a) is a rearticulation of the second part, on technoscience. The central thesis of the dissertation is that twentieth-century philosophy is characterized by an increasing preoccupation with language. The diagnosis of this preoccupation is what Hottois calls his ‘metaphilosophy’: “Since we are looking for the forms and causes of linguistic haunting, our essay is by no means a new philosophy of language: it is a reflection on the philosophical inflation of language in all its forms; it is a metaphilosophy of language.” (Hottois, 1981, p. 20)^1^

This diagnosis is not new and has been canonized as the ‘linguistic turn’ (Rorty, 1970). But Hottois is interested in the linguistic turn not only because of its ubiquity, but also because of what he sees as its fundamental limitations. “This essay on the metaphilosophy of language,” Hottois writes, “attempts to break out of this omnilinguistic space.” (Hottois, 1981, p. 7) In his own terminology, Hottois defines this as *secondarity* (Hottois, 1980). For Hottois, language has two fundamental properties: secondarity and referentiality. While the latter refers to how language can refer to a non-linguistic object, the former refers to its ability to speak about language itself, for example through a metalanguage. Although the history of philosophy has always been concerned with both elements, with referentiality often being the dominant one, within contemporary philosophy the focus has shifted to secondarity (Hottois, 1981, p. 23).

In addition to this new terminology, I argue that Hottois’s metaphilosophy of language introduces two innovations. The first is his distinction between two modes in which this inflation of language has taken place: *metalinguistics* and *adlinguistics*. While the former refers to what has happened within analytic philosophy, the latter refers to continental philosophy.

Metalinguistic philosophy refers to philosophy that is not concerned with the traditional referential project of a metaphysics of analyzing the world, but with analyzing discourses about the world. For example, logical positivists such as Rudolf Carnap and ordinary language philosophers such as J. L. Austin are concerned not with nature but with what scientists say about nature. As these examples indicate, metalinguistic philosophy can become a form of ‘linguisticism’, in which all philosophical problems become problems of language. In light of his first book, one can also refer to the case of Ludwig Wittgenstein. However, Hottois qualifies that although the early Wittgenstein of the *Tractatus* is a metalinguistic philosopher, the later Wittgenstein of the *Philosophical Investigations* must be seen as an adlinguistic philosopher (Hottois, 1981, p. 66).

Adlinguistic philosophy is characterized, not by a shift from the referent to an analysis of the discourse about the referent, but rather by an implosion between referent and discourse: the idea of a referent outside language loses its meaning, and the world itself must be understood linguistically. “Adlinguistic secondarity […] has a non-referential view of the nature and origin of linguistic meaning: meaning emerges between words, it is the product of a structure in which differences and similarities come into play and which is only realized in the sentence, the discourse or the text” (Hottois, 1981, p. 22). Here Hottois refers to traditions such as phenomenology (Martin Heidegger, Maurice Merleau-Ponty), hermeneutics (Hans-Georg Gadamer) and (post)structuralism (Jacques Lacan, Jacques Derrida).

The case of (post)structuralism is the most uncontroversial example, since this tradition is concerned with the analysis of the structures of discourse. Indeed, Goffi (1988) suggests that for Hottois the distinction between metalinguistics and adlinguistics can be traced back to the split between the two main theories of meaning in twentieth-century philosophy. Whereas metalinguistic philosophy goes back to Gottlob Frege’s distinction between sense and reference, adlinguistic philosophy relies on Ferdinand de Saussure’s distinction between signifier and signified. The former leaves the traditional notion of reference intact while opening up a new realm of discourse for philosophers to analyze, while the latter tends to problematize the function of referentiality. But Hottois also argues that earlier movements, such as phenomenology and hermeneutics, are forms of adlinguistics. Although they seem to move from concepts to ‘the things themselves’, they often do so by reinterpreting the object of experience, or Being in general, as something to be interpreted and read, and thus ultimately taking the form of a discourse. Given this tendency to identify Being and Logos, a similar implosion of the distinction between reference and meaning takes place (Hottois, 1981, 12–13).

Hottois’s second innovation concerns his explanation of why this inflation of language took place. He offers two hypotheses, linked to the distinction between metalinguistics and adlinguistics:

Our guiding hypothesis is twofold. To a considerable extent, the reduction of philosophy to language consists in a simple shift of reference (from ontology to linguistics), a consequence of the monopoly of science and technology on extralinguistic reference. On the other hand, the inflation of language, especially on the continent, is also the manifestation of a deeper and more subtle reaction of philosophy to the practical and theoretical undermining, in the most advanced techno-sciences, of the unprecedented, specific definition of man as an essentially speaking living being, a definition that has permeated philosophy from the very beginning. (Hottois, 1981, p. 8)

In other words, metalinguistic philosophy is the product of a shift in the authority to speak about the referent: whereas metaphysics used to claim this authority, it has recently shifted to the sciences. “The retreat into language is the consequence of the referential monopoly acquired by the sciences. This scientific headlock on referential logos condemns philosophy to a descriptive or critical engagement with (natural, scientific and technical) discourses and languages.” (Hottois, 1981, p. 138) However, for Hottois this shift in authority goes hand in hand with an inability to acknowledge and articulate the specificity of the technical dimension of the sciences, or in other words: technoscience. Metalinguistic philosophy recognizes how science has become more important, but interprets the relation of science to the world in linguistic terms: science aims to produce an adequate linguistic representation of the world. As Hottois notes, within metalinguistic philosophy science “extends, but in a more adequate, certain and differentiated way, the ontological intention of producing a linguistic object through which man would master the universe in the contemplative mode.” (Hottois, 1981, p. 138) This explains why metalinguistic philosophy tends to see this shift in authority as unproblematic, and even welcomes it. “If the philosopher of metalinguistic secondarity has so readily accommodated himself to the imperialism of the sciences, it is probably because he has failed to perceive the extent to which contemporary technoscience breaks with the traditional essence of man on which philosophy, and especially ontology, is founded.” (Hottois, 1981, p. 139).

The situation is different for adlinguistic philosophy, which recognizes that science is something that cannot be reduced to language, but goes beyond it. “*The perception of an - at least partial - incommensurability between techno-science and traditional philosophy* […] *seems to lie at the roots of continental secondarity*.” (Hottois, 1981, p. 139). The result is a critical engagement with science, especially its technical dimensions. The archetype of this is Martin Heidegger, whose *The Question Concerning Technology* redefines modern science as technological and a fundamental threat to proper thinking. In this sense, adlinguistic philosophy has a fundamentally ambivalent attitude towards technoscience: the technological dimension of science is recognized, but as something that must be overcome in order to save philosophy, which is itself defined as a linguistic activity. “*Continental secondarity is the complex product of the encounter between a very powerful ontological ambition* […] *and an extreme sensitivity to everything in the techno-scientific cosmos* […] *that undermines this Logos and undermines the image of man as the bearer of this Logos and protected by it.*” (Hottois, 1981, p. 155).

On this point, adlinguistic philosophy is as limited as metalinguistic philosophy: its focus on language makes it incapable of recognizing the specificity of technology in contemporary science. It is in this context that Hottois introduces the concept of ‘technoscience’ in order to speak about science in a way that recognizes this non-linguistic, technical dimension:

the term ‘techno-science’ seems to us both convenient and elegant to designate the contemporary technical and scientific fact whose specificity (in relation to modern and positive science) lies precisely in the absolutely inescapable character (including and central to so-called ‘theoretical’ research) of technical manipulation, construction and invention (in all fields: psychology, biology, physics…). (Hottois, 1981, p. 157)

Hottois ends the book with a plea for an alternative to the inflation of language. The way out, for Hottois, is not a return to traditional metaphysics, which remained linguistic. A ‘post-secondary’ philosophy requires a confrontation with

the root cause of its linguistic neurosis or psychosis: the techno-scientific universe, which philosophical thought has so far either purely and simply excluded from its field of consciousness, or tried to assimilate and divert by maneuvers that have only succeeded in making the imprisonment in language more sophisticated and deeper. (Hottois, 1981, p. 160)

In other words, a post-secondary philosophy requires us to develop a proper philosophy of technoscience: a philosophy capable of acknowledging and recognizing the non-linguistic relations we have with the world. “At the center of post-secondary attention should be *these new non-linguistic and non-theoretical bridges* that are active in all current techno-scientific advances. As concrete operations, these bridges are *interventions*.” (Hottois, 1981, p. 161).

## A Philosophy of Technoscience

Hottois’s metaphilosophy of language demonstrated the inability of contemporary philosophy to conceptualize technology because it clings to a traditional conception of philosophy as concerned with language, symbols, and meaning. If it conceptualizes science at all, it tends to reduce it to contemplation, discourse, and representation. The association of science with these three activities - thinking, speaking and seeing - mainly implies a passive image of science, where science is only concerned with creating an adequate picture of reality.

In his philosophy of technoscience, Hottois aims to offer a description of science that does justice to the intuition that science not only describes the world, but also changes it. To achieve this, Hottois focuses on the technical side of science. An articulation of this concept of technoscience can be found in the last part of his dissertation, but even more prominently in later publications such as *Le signe et la technique* (1984a), *Pour une éthique dans un univers technicien* (1984b) and later in *Entre symboles et technosciences*. In this context, Hottois sketches a picture of science that echoes what has been called the ‘practice turn’ in philosophy of science of the 1980s (Soler et al., 2014; Potters & Simons, 2023). We therefore find in Hottois claims that could have been formulated by Ian Hacking, Don Ihde or Bruno Latour:

Contemporary science is not primarily, let alone exclusively, a set of texts and representations. It is a shifting complex of machines, networks, operations, powers and systems; it is a set of actions, processes and procedures that enable us to intervene in nature and in the human condition in a way that is radically different [from symbols…]. (Hottois, 1996a, p. 13)

A simple equation of Hottois with the practice turn, however, loses sight of both the specific way in which Hottois defines technoscience as well as his main motivation for doing so. I will return to Hottois’s motivation later, but first I want to articulate the specificity of his diagnosis of technoscience.

In the introduction, we pointed out a peculiarity of Hottois’s practice turn, namely that he arrives at his conceptualization of technoscience, not on the basis of a series of empirical case studies of scientific practices, but through a method of speculation inspired in particular by science fiction (Hottois, 2000, 2022). As Jean-Nöel Missa (2022, 10) notes, “the young Hottois had been enthusiastic about science fiction since childhood, before he began his studies”. This interest formed the foundation of Hottois’s fascination with technology. His books contain numerous references to science fiction writers such as Arthur C. Clarke and Stanislaw Lem, as well as to futurologists such as Vance Packard, Jöel de Rosnay, François de Closets and Alvin Toffler. In unpublished notes, Hottois also conceptualized his own career as deeply intertwined with science fiction (SF): “Chronology: 50 and 60: SF and novels; 70: philosophy; 80: SF, philosophy and bioethics; 90: bioethics; 2000-: a loop of philosophy and bioethics in the light of an integral SF.” (quoted in Missa, 2022, 8n1) In 1981, Hottois even wrote a science fiction novel, *Species Technica*, although he would not publish it until twenty years later (Hottois, 2002).

In Hottois, we thus find an alternative method, based on the analysis of speculative scenarios, as well as a discourse analysis of science fiction novels, futurology books, science policy reports, and other documents, which aims to diagnose the current state of science as well as its challenges through the concept of technoscience. Such speculative methods are nowadays often criticized (e.g. Nordmann, 2007), but this article will argue that Hottois offers us a case where these methods can be productive. For instance, although he himself never systematized it in this way, I want to propose that we can derive eight characteristics of technoscience from his work.

For Hottois, the first and most important characteristic of technoscience is its *operationality*. Technoscience is fundamentally non-representational - aiming to capture reality through symbols - but operational - aiming to operate and intervene in the world. For Hottois, this operationality has two dimensions:

In reality, the double operative key of techno-science combines the *technicization of experience* and *mathematization*. Both are alien to the alliance of symbol and gaze […] and distance man from his symbolic sojourn in the universe. The technicization of experience corresponds to the substitution of sensitive natural experience, which was like a respectful reading of things, by technically and mathematically structured experimentation, which provokes events. (Hottois, 1984a, p. 63)

While experimentation refers to the use of technical instruments and environments to produce and manipulate phenomena, mathematization refers to how the theoretical component of this technoscientific research often relies on mathematical operations that, while effective, are too complex for a single individual to understand, let alone link to specific symbolic images. Instead, they are “blind symbols that reject all intuition”. For instance, “the equivalence between matter and energy in no way tells us what matter is or what the nature of energy is: it merely indicates certain transformations.” (Hottois, 1984a, p. 64).

The second and third characteristics are “the *indissolubility* of the two poles of the theoretical and the technical-operational; and the *ultimate primacy* of technique over theoria.” (Hottois, 1984a, p. 60) In technoscience *science and technology*, discovery and invention, tend to *become inseparable*. But technoscience also implies a shift from the primacy of theory to the *primacy of the technical*, of praxis, with the latter “as the source, means and aim of contemporary scientific research.” (Hottois, 1984a, p. 60) Theory becomes an instrument of technical intervention:

The nature or structure of theory becomes increasingly exclusively mathematical, while the correlates of residual concepts are no longer referential objects but groups of operations, so that the ‘real’ we are aiming for is no longer ontological but itself operative: *the real is that which is (re)producible*,* manipulable*,* transformable*,* and no longer that which is visible*,* intelligible or comprehensible*. (Hottois, 1984a, 61–62)^2^

At first glance, these two characteristics seem to be in tension: how can the technical gain primacy and at the same time be indistinguishable from the theoretical? Here it is important to distinguish indistinguishability from indissolubility. For Hottois, the technical and the theoretical are still two separate things, even if in practice they can never be separated. At the same time, it is the technical-operational side that dominates: “the theoretical remains active in the research process, but it is only a moment in it - that of the construction of a model, which, like a plan, is a tool of symbolic representation at the service of practical, physical steps - or it is a springboard for new, active investigations” (Hottois, 1984b, p. 11). This does not mean, however, that science is reduced to technology or merely serves practical and economic incentives. For Hottois, the primacy of the technical-operational “does not mean […] that science is in bondage to the technical aims of its application, and that in this sense it would become exclusively utilitarian and self-interested. That science is technicist means first and foremost that the relationship of science to reality is essentially mediated by technology.” (Hottois, 1984b, p. 12).

A fourth characteristic is the *ontological silence* of technoscience: in contrast to the symbolic image of science, technoscience does not pretend to produce an ontological discourse about reality:

Humanity cannot relate in a cultural-natural way to processes that it encounters and will encounter only technically and mathematically. *Hence the great ontological silence induced by contemporary physics.* This silence is merely the logotheoretical expression […] of the purely operative (blind and mute) nature of techno-science. The operative encounter is not a possible source of ontology precisely because it is operative, i.e. non-linguistic and non-theoretical. (Hottois, 1984a, p. 70)

This is related to the fifth characteristic, the *different object* of technoscience. Whereas the symbolic image of science has as its correlate the ‘given’ object, with its essence to be revealed, technoscience is concerned with the potentialities of objects. “Generally speaking, the correlate of traditional science or theoretical knowledge was the essence of the object to be known; the correlate of techno-science is the *plasticity of the object to be manipulated* (whether physical, living or thinking matter). Being was the correlate of seeing and speaking. The possible is the correlate of doing.” (Hottois, 1984b, p. 12).

For Hottois, this is also reflected in an *anti-theoretical principle* that accompanies technoscience, which he often summarizes as: *everything is possible*. For technoscience, there are no a priori epistemic restrictions on what can be explored and created - the restrictions are only practical, not theoretical. “A principal function of any theory, and an essential aim of any theoretical project, is to *determine*, that is, to set a framework, a set of limits (by indicating what is, or what is possible and what is not, on the basis of what is), and to predict. In this sense, the technical principle of ‘everything is possible’ can rightly be described as absolutely anti-theoretical.” (Hottois, 1984a, p. 145)^3^

Linked to this fifth characteristic, the sixth refers to *another criterion of success*. Whereas science is judged by the extent to which it faithfully represents reality, technoscience aims to operate and manipulate that reality. “Technoscience is measured by its essentially silent physical productivity and operativity, not by any discursive, symbolic or speculative richness and creativity.” (Hottois, 1993, 22) The criterion of a successful technoscientific project lies in its *effectiveness*: to what extent was the project able to operate and manipulate that which it sought to transform?

Through this question we can already see how technoscience opens the door to practical questions of its interaction with society. This can also be seen in the last two characteristics of technoscience. The seventh is the dynamic nature of technoscience: technoscience is to be understood as a *creative process*. Technoscience is constantly changing and, in the process, affects its environment. In this sense, Hottois’s diagnosis of technoscience goes beyond that of other philosophers who stress the role of technology and experimentation in science, such as Bachelard (1934) or Hacking (1983). Hottois is often sympathetic to their views, but feels that they are not radical enough. Both Bachelard and Hacking will emphasize how crucial technology in the form of instruments is to science. But this seventh characteristic shows how, for Hottois, the centrality of technology in technoscience goes much further than the epistemology of instrumentation, and also evokes a different anthropology and ontology.

Finally, there is the *intrinsic connection between technoscience and social*,* political*,* economic and ethical concerns*: “while theoretical science could be said to be pure and innocent, techno-science, since it is essentially an activity that modifies the world, is never enitrely innocent. As a praxis, it is ethically problematic.” (Hottois, 1984b, p. 12) The rest of this article will argue that this seventh characteristic, that technoscience is a temporal process, is crucial to Hottois’s understanding of technoscience, while the eighth characteristic, the ethical concerns that flow from it, is at the center of Hottois’s turn to bioethics.

## The Temporality of Technoscience

Within the picture of technoscience sketched above, there remains a fundamental ambiguity. In his diagnosis, Hottois often oscillates between an *essentialist thesis* - science has always been technoscience - and a *historical thesis* - science became technoscience in modernity. For example, in his textbook *De la Renaissance à la Postmodernité* (1997), Hottois writes that “*ancient science was logotheoretical*, i.e. constituted by language (logos) and an intellectual or spiritual insight (theoria).” (Hottois, 1997, p. 50) It was only with Francis Bacon that modern science became “*active*,* operational*,* effective* rather than contemplative and verbal. […] In short, an image of nature emerges that is fundamentally manipulable, transformable, subject to human exploitation and reconstruction. We are here at the antithesis of the ancient logotheoretical science.” (Hottois, 1997, p. 56)^4^

This ambiguity is linked to a fundamental opposition between symbol and technique that runs through Hottois’s work. For Hottois, this is an ontological assertion: the world consists of at least two different things, symbols and techniques, which cannot be reduced to each other. The previous sections have discussed features that illustrate this opposition. Hottois associates symbols with language, representation, vision and thought. Technique, on the other hand, is what runs the risk of being ignored in philosophy. Describing technique in its own terms is more difficult, precisely because it refers to that which eludes symbolization. In this sense, the main concepts associated with technique by Hottois tend to be open and negative. Technique is associated with creativity, opacity and openness (whereas symbols tend to be static, comprehensible and closed). Technique refers to the physical, non-symbolic processes that take place in the world and that express themselves in, among other things, technical processes.

Hottois associates technique with a transcendence, but different from a symbolic transcendence, which “postulate[s] that man belongs to a supernature in which he participates thanks to the spiritual component of his essence” (Hottois, 1999, p. 177). Hottois initially calls this alternative form of transcendence a “Cosmic Wall” (*Mur Cosmique*) (Hottois, 1979). Later on, he speaks of “dark transcendence” (Hottois, 1984a, p. 152) or “operational transcendence” (Hottois, 1999, p. 177). As he explains in *Le signe et le technique*: “*‘Dark transcendence’ is a metaphysical metaphor* […] *for the fundamental experience of the primacy of the operational*. This experience […] is that of the opacity and limitless openness of the future.” (Hottois, 1984a, p. 158) For Hottois, dark transcendence does not refer to a specific symbolization of technique, but testifies to “the immediate and absolute opacity that comes over the voice and the gaze when one tries to think seriously about the primacy of the operational-technical.” (Hottois, 1984a, p. 186).

However, it is important to qualify this opposition between symbols and techniques. First of all, it does not imply a simple equation between technoscience and technique. The point is not to reduce technoscience to technique, but to recognize both dimensions at work in technoscience. For Hottois, “technosciences are never ‘pure’: they are always accompanied by and invested with symbols that advance, slow down and guide them.” (Hottois, 1996a, p. 93) Another way of making this point is to distinguish the opposition between symbol and technique from that between *theoria* and *praxis*. While at first glance they seem similar, a better way to understand what Hottois is up to is to say that he is arguing for the primacy of *praxis* over *theoria* (as we discussed in the last section), where *praxis* (and *theoria*) consists of both symbol and technique. Joseph Rouse articulates this point very well in relation to the aim of philosophy of experimentation in general: “Even theory is more practical and artisanal than philosophers usually realize. The problem, then, is not the neglect of one part of science (experiment) in favor of another (theory), but rather a distorted perspective on the scientific enterprise as a whole” (Rouse, 1987, xi). Thus, a reconceptualization of science as *praxis* rather than *theoria* is a way of giving both symbol and technique their proper place in science.

In fact, it is their interaction that also helps to make sense of the history of technoscience: while new technical developments have symbolic effects – through imaginaries and ideologies -, existing symbols in turn guide technoscientific research. Hottois conceptualizes their interaction as a technosymbolic feedback loop, a theme that will be taken up later in Dutch philosophy of technology:

man modifies the environment technically; he relates symbolically to this produced environment, i.e. he reshapes himself symbolically, individually and collectively, by evaluating it culturally: from this cultural-symbolic evolution, which redistributes values and meanings, desires and fears, man returns to the technophysical environment and continues its elaboration in a specific direction. And so on. (Hottois, 1996a, p. 176)

For Hottois, this interaction between these two fundamentally different things causes confusion among philosophers. First, their properties are often confused, for example by falsely attributing technical power to symbols, as happens in magic or the talking cure of psychoanalysis. Conversely, symbolic power is often attributed to techniques, as in vulgar forms of Marxism where culture is a mere product of the base structure, or in superficial forms of neuropsychology that reduce the mind to the brain.

But there is often a second, more crucial confusion at work, namely that of reduction. Again, this can run in both directions. Earlier I already described an instance of the reduction of technique to symbols, which happens in the symbolic image of science. This is the main fault of linguistic philosophy: its reduction of technique to symbols. “*The denial of the irreducible difference between the realm of the technical and the symbolic order*,* the blindness to the irreducible character of technique*,* is the constitutive illusion of twentieth-century philosophy*.” (Hottois, 1984a, p. 115) But symbols can also be reduced to the technical. Symbols are then seen as mere tools for the installation of action. Hottois sees this at work in rhetoric, where language is seen as a mere means to an end, as well as in Leibniz’s project of *mathesis universalis* (Hottois, 1990a). But more problematically, he sees it at work in certain technoscientific research programs and technocratic forms of politics, where human culture is reduced to mere techniques to be manipulated at will, irrespective of human symbols and affections. “Human language is in a sense *disaffected*.” (Hottois, 1996a, p. 92) It is on this point, and this point alone, that Hottois supports the often very critical use of the concept of technoscience by French philosophers (see Sebbah, 2010), in particular Michel Henry’s *La barbarie* (1987). For Hottois, the negative connotations of technoscience as a barbaric destruction of human culture are not inherent to technoscience, but characterize technoscience under the spell of an ideology that reduces symbols to technique.

These remarks also help to understand the philosophical program behind Hottois’s opposition between symbols and technique. At first glance, this opposition looks similar to a popular theme in critical theory, namely the critique of instrumental rationality (Benhabib, 1986; Schecter, 2010), which is often associated with technology (Feenberg, 2018). In a nutshell, it entails a critique of how the lifeworld is reduced to, or colonized by, instrumental reason, where the instrumental means have become an end in themselves (Habermas, 1987). But the above dissociation from Henry’s claim suggests that Hottois is aiming at something else: technique must not be equated with instrumental rationality, which is merely an ideology, an attempt to symbolize technique. To accept instrumental rationality as the main enemy would again be to reduce techniques to symbols.

My proposal is instead to read Hottois in critical dialogue with French philosophy of technology (Loeve et al., 2018), in particular with what is often called the biological philosophy of technology (Clarizio, 2021). This tradition is often associated with Henri Bergson, André Leroi-Gourhan, Georges Canguilhem, Gilbert Simondon and Bernard Stiegler. Hottois, however, is never mentioned in this context. But there are good reasons for including him. Not only does he regularly refer to Bergson, Leroi-Gourhan and Canguilhem, he also wrote the first book on Gilbert Simondon (Hottois, 1993) and supervised one of the first PhDs on Simondon's work, Chabot (2003).

As the name suggests, biological philosophy of technology is associated with the use of biological metaphors and analogies to understand technology and its history (Clarizio, 2021, 2023; Feyles, 2022). We also find these biological analogies in the work of Hottois (e.g. Hottois, 1996a, p. 100). I do not want to suggest, however, that Hottois advocates a philosophy of technology that places life at the center, that sees technology, for example, as the expression of an *élan vital*. In fact, Hottois often seems rather skeptical of attempts to push biological analogies too far. Instead, my association of Hottois with this tradition can be understood in at least three alternative ways.

A first, and less binding, reading is that the link between the two is merely heuristic, didactically allowing me to bring to the fore a set of analogies and problems that would be harder to emphasize if Hottois were placed in relation to critical theory or the Dutch empirical turn. On this reading, the connection may be entirely fictitious, but useful. A second, stronger reading is that the connection is not one of equation but of discussion. To link Hottois to this tradition, then, is not to say that they (fundamentally) agree, but that Hottois often defines itself in critical dialogue with this tradition, even when they disagree.

A third, and perhaps the most controversial reading is that Hottois and the French biological philosophy of technology can be positively linked, but only because his work offers a new prism through which to re-read this whole tradition. This reading argues that, beneath these biological analogies, there is another theme characteristic of this tradition that is crucial to Hottois’s philosophy of technoscience. French biological philosophy of technology, according to this reading, is first and foremost a *philosophy of temporalities*. This comes as no surprise to scholars familiar with Bergson or Stiegler, who are often seen as thinkers of time (e.g. Stiegler 1993; Allen, 2023). Bergson is famous for his critique of how concepts reduce the experienced durations of the world to static symbols in function of the pragmatic goals of life. Similarly, Leroi-Gourhan and Simondon can be read as thinkers who seek to understand technical artefacts and machines primarily through their temporalities: to understand technologies, we must resist static machine metaphors and think through their genesis and evolution. Just as Bergson argues that mechanistic metaphors cannot capture the durations of life, Leroi-Gourhan and Simondon argue that they cannot capture the durations of technology. In a paradoxical way, mechanistic metaphors are unsuited for machines since these metaphors are not designed to understand them, only to deal with them pragmatically. In this third reading Hottois’s work can be read in a similar way, namely as a thinker of the temporality of technoscience who resists the conflation of the durations of technoscience with static symbols introduced to deal with science pragmatically.

The claims made in the rest of the paper are in fact compatible with all three readings. We therefore need not decide here which one is correct, especially since the justification of the strong reading would require more substantial evidence and analysis. But whichever reading we follow, in drawing the connection between Hottois and this tradition, speaking of a ‘biological’ philosophy of technology may in part be a misnomer, since what is actually essential in linking the two is their focus on temporality.

We also find such a tension between biology and temporality in Hottois. In his work, biology plays a crucial role, but because it introduces us to “a radically dis-anthropocentric temporality” (Hottois, 1996a, p. 83). Alongside one’s *experienced history*, and *human history*, there is the ‘realist postulate’ of *cosmic time*:

To believe that it was this immensely long process that led locally to the appearance of living human beings, to their social organization, to their technical and symbolic productions […]. To believe - and perhaps more importantly! - that there is still an immensely long time in the future, and that cosmic processes (with the possible production of life forms) will continue for billions of years, whether or not humanity continues to exist during that time. (Hottois, 1996b, p. 175)

20th century linguistic philosophy negates this postulate, for example in phenomenology. “For phenomenological consciousness, cosmic temporality is an extremely derivative notion, a distant product of the symbolic institution of mathematical quantification and modern science.” (Hottois, 1996b, p. 175) Such an anthropocentric stance, however, fails to take the temporality of technology seriously. “The philosophy of technoscience must take into account the temporality of technoscience.” (Hottois, 1999, p. 28).

Hottois conceptualizes this difference between human history and cosmic time through the concepts of *prehistory* and *posthistory*. The technosciences have opened up these two novel temporalities. Prehistory, which refers to the period before written sources, only makes sense through technoscientific research, which reconstructs this past on the basis of biophysical evidence. “It is techno-science that teaches us that man is the product of a game of physical chance, of mutational manipulation, of a biochemical combinatorial process that no guiding gaze or logos could overlook.” (Hottois, 1984a, p. 38) It is by analogy with this highly contingent and open cosmic evolution, revealed technoscientifically through prehistory, that Hottois speaks of posthistory, referring to the distant future, a temporality in which our historical narratives make no sense:

The evolutionist perspective tends to see history as an extraordinarily short, undeniably specifically human moment, preceded by an extraordinarily long prehistory and possibly followed by a ‘posthistorical’ evolution, of which we can anticipate nothing, because the future is perceived today as both open (the indefiniteness of the possible) and opaque (the impenetrability of any anticipation once we move even a few decades away from the present). (Hottois, 1988, 92–93)

This openness and opacity is a property of the temporality of the technosciences themselves. Hottois also speaks of *technochronies*, as a technoscientific replacement of symbolic chronologies:

Techno-science confronts us with *technochronies* that are out of all proportion to the human experience of time - the incomparable brevity of microphysical time (the life of certain particles) and the questioning of irreversibility, of the vectorial sense of time that sustains our existence; Relativistic phenomena of temporal contraction and petrifaction - the supposed limit of the temporality of ‘black holes’; the immensity of geological and cosmological durations; the operational occupation of duration by computers to an incomparably greater degree than is possible with human activity (in a few seconds, a computer performs a series of operations that would take an individual years to complete). *Operational mediations* - technical and mathematical - *are the only way for us to gain access to all these technochronies* which we cannot experience directly and which therefore *elude any symbolization*. *Technochronies* and the chrono-logies of individual and collective history are *incommensurable*. The former constitute neither events nor historical durations. (Hottois, 1984a, p. 78)

Technoscience does not operate within human symbolic categories, but must be understood as the continuation in time of a “cosmic creativity” of which human history is only a localized period, while this creative process will continue long after human history has ended. “It is this creativity that produced man; it is this creativity that pervades him and makes him a technocosmic generator; it is this creativity that launched mankind into history […]; it is this creativity that pushes him into the post-historicity of technochrony.” (Hottois, 1984a, 128–129) Technoscience illustrates the situatedness of humanity, which is merely “a product of cosmic operativity that does not presuppose a conscious and willing subject. [….] It is man who becomes a moment and an instrument of creative evolution, the present form of which is techno-science; it is not techno-science that is the instrument of the fulfilment of man’s essence or nature.” (Hottois, 1984b, p. 42) It is in this sense that Hottois spoke of a Cosmic Wall, a dark transcendence, and an operational transcendence. “The transcendence of the Future is called ‘dark’ because it is impenetrable, unpredictable.” (Hottois, 1984a, p. 160)^5^

From this perspective, we can begin to understand Hottois’s fascination with the question with which we began this article: *What will happen to humanity in a million years?* For Hottois, this question does not seek a specific answer, but confronts us with the temporality of technoscience, which “transcends any symbolic or theological ordering of time” (Hottois, 1996a, p. 211). Or, to quote Hottois more fully:

What will become of man in a million or ten million years? This is a legitimate and deeply *philosophical* question. It is not a scientific or technical question that can be resolved methodically. […] The question posed is profoundly philosophical *because it has no answer*, because it gives rise to an unprecedented experience of temporality, an experience that nevertheless corresponds to techno-scientific time: *the experience of a radical opacity and an unlimited openness of time.* (Hottois, 1984a, p. 83)

Moreover, it helps to explain Hottois’s fascination with both transhumanism (Hottois, 2017) and science fiction (Hottois, 2022). What fascinates Hottois in both phenomena is their indirect recognition of the radical openness and opacity of cosmic time. In other words, good science fiction does not convince the reader of a particular future scenario, but confronts the reader with the radical opacity of the future. Similarly, transhumanist thought is powerful to the extent that it shows the contingency of the symbolic forms by which we understand the human today. But to the extent that transhumanist thought imagines itself capable of adequately predicting the long-term future of humanity, it is just another instance of the attempt to reduce technique to symbols. Hottois’s conclusion from cosmic time seems in that sense to be the opposite of that of many transhumanists today. The conclusion is not so much that we now have the power and knowledge to shape our future, but that they are fundamentally unattainable. Instead, Hottois concludes with a certain prudence: “we - humanity - *have all the time in the world*. It is neither wise nor prudent to rush into things, as if tomorrow we could already become ‘like gods’. Utopianism is an essential driving force for the ongoing invention of the future, but when it asserts itself in the short term, it is obtuse and dangerous.” (Hottois, 1999, p. 29).

## From Technoscience to Bioethics

In articulating the temporality of technoscience, Hottois often emphasizes how it leads to “*desymbolisation*” (Hottois, 1996a, p. 81). Technoscience operationally destabilizes or destroys symbolizations. We see this at work in bioethical questions, concerning birth, reproduction and death: technoscience allows us to do things that do not make sense in our symbolic frameworks (Swierstra et al., 2009: Simons, 2022b). In this context, Hottois also emphasizes a second consequence of this temporality: “*de-ethicisation*” (Hottois, 1996a, p. 82). In his early work, Hottois seems very skeptical about whether the challenge of technoscience can be met through ethics. The argument seems simple: ethics resides on the side of symbols, and imposing an ethical framework on technoscientific research entails the closure of the openness of technoscience in favor of the contingent present. In this sense, desymbolisation seems to go hand in hand with de-ethicisation. This was the argument in his dissertation (Hottois, 1979, 362–368), which he still endorsed twenty years later (Hottois, 1999, p. 20).

At the same time, his philosophy of technoscience offers a philosophical argument for the necessity of ethical engagement with technoscience: the technical side of technoscience demands ethical reflection. We return here to the main motivation for Hottois’s ‘practice turn’: the technical side of science must be emphasized in order to be able to fully articulate and evaluate the ethical challenges that technoscience poses in our contemporary society. Technoscience leads to bioethics because technoscience is fundamentally *operational* and intervenes in the world: “while theoretical science could be said to be pure and innocent, techno-science, being essentially a world-altering activity, is never entirely innocent. As a practice, it is ethically problematic.” (Hottois, 1984b, p. 12) Since technoscience always changes the world technically, we need to think about what changes are ethically acceptable.^6^  

But how to combine this need for ethical reflection (due to the operational nature of technoscience) with its fundamental impossibility (due to the opacity of this operationality)? Hottois found a way out of this dilemma through his systematic study of the work of Gilbert Simondon, which led to his concept of ‘guidance’ (*accompagnement*):

With the help of Simondonian thinking, the notion of guidance took shape. To symbolically guide the technosciences is what Simondon tried to do in his awareness of the difference between symbol and technique, and of the - moral - duty to bear this difference without subordinating one of their terms to the other. (Hottois, 1996a, p. 18)

Hottois puts this idea into practice, mainly through opportunities in the 1980s to engage with the emerging field of bioethics. In particular, the then Belgian Secretary of State for Health and Disability Policy, Wivina Demeester, organized a national symposium on ‘Bioethics in the 1990s’ (Demeester, 1987). While it was easy to find Catholic representatives, it was more difficult to find secular counterparts. Hottois had published *Pour une éthique dans un univers technicien* (1984b) and was therefore invited. That same year, together with the anthropologist Charles Susanne, Hottois founded the *Centre for Interdisciplinary Research in Bioethics* (CRIB) at the ULB. Over the next decade, Hottois also became an active member of the Belgian National Bioethics Committee and the European Group on Ethics in Science and New Technologies (EGE). This led to a number of joint publications on bioethics (e.g. Hottois & Parizeau, 1993; Hottois and Missa 2002).

But what does this concept of ‘guidance’ entail? For Hottois, it suggests a novel task for ethics, namely that it should ‘guide’ technoscience without introducing static symbolic constraints. “Guiding does not mean anticipating, or preceding, or founding” (Hottois, 1996a, p. 204). In a sense, ethics has always been guiding technoscience, but without recognizing the temporality of technoscience, resulting in failed attempts to control technoscience through fixed symbolic systems. The task of philosophical guidance, for Hottois, is to systematize these spontaneous attempts, to point out their limits and problems. If ethics wants to limit and control technoscience, we must always ask: “control in the name of what and who? In the name of what symbol, of what capital name, can we legitimately subordinate the technosciences […]?” (Hottois, 1996a, p. 14). Any attempt to limit technoscience by ontological appeals to ‘human dignity’ or ‘nature’ are out of the question. They tend to petrify and absolutize a contingent historical state of (human) nature, whereas in the light of the temporality of technoscience (human) nature is a creative and open process: “The knowledge we have of biocosmic evolution and human history renders particularly implausible, and therefore symbolically inoperative, the images and notions of natural order, harmony, stability and finality, or of an a priori meaning given in nature, that fuel ontological arguments.” (Hottois, 1999, pp.79–80).

But what does such a philosophical guidance look like? First of all, ethics must move from an a priori and static activity to an *a posteriori* and dynamic activity. It must recognize the temporality of technoscience in order to “allow technosciences to develop their emancipatory dynamic freely, without being subordinated to dogmatic symbols that limit research a priori.” (Hottois, 1996a, p. 20) In his work, Hottois often captures this through the terminology of *openness* and *closedness*, a terminology that can be traced back to Simondon (1958) and Bergson (1932). However, it would be wrong to read Hottois’s work as a mere plea for the removal of all symbolic barriers to technoscientific development. Instead, Hottois argues for a middle ground between total openness and total closure, since both lead to destruction. Hottois seems to draw on another biological analogy: the ideal state of an organism is neither total closure nor total openness. Both would lead to death, either through stagnation or destruction. This is a lesson that Hottois learns from Simondon:

This fault or error […] has two faces: that of massive identification, which unites everything in an immovable totalitarian symbol (which may be an instituted religion, an ideology, a technocracy, etc.), and that of radical differentiation, which abruptly isolates, irreparably dissociates, and breaks every bridge with the other (Hottois, 1993, p.47).

Hottois also often captures this through another Simondonian concept, that of *metastability*: a state in a dynamic system where there is a local and temporal form of stability, but which will shift to another state in the future. Similarly, in the case of the governance of technoscience, we would need “a symbolization capable of perpetual, metastable, and evolutionary adaptation” (Hottois, 1999, p. 76).

In his own terminology, Hottois often frames it in terms of symbols and techniques. The ideal guidance of technoscience recognizes that both should play a role, and the danger is that one side becomes dominant:

As Gilbert Simondon has pointed out, what seems crucial in terms of the interaction between symbols and technoscience is to maintain a relationship of freedom with respect to both technique and symbol. This means that if the symbol cannot enslave technoscience, neither can technoscience enslave the symbol. The first form of domination leads to theocracy, the second to technocracy. (Hottois, 1999, p. 177)

In other work he captures this through a distinction between two different forms of alienation. On the one hand, there is the familiar *socio-cultural alienation*, where people are alienated by an oppressive ideology. But, there is also *biophysical alienation*, when technoscientific developments lose all connection with existing symbolic structures. This is particularly the case when technoscientific developments threaten to alter the most basic human capacities, such as the capacity for speech, free choice, ethical sensibility (Hottois, 1988, 94–95). Philosophical guidance steers between these two extremes, exchanging an ontological view of symbolic distinctions for a pragmatic one. “Yes to moratoria, debates, discussions, slowness and prudence. No to excommunications, ostracisms, dogmatic and absolute rulings and condemnations, motivated by transcendental references that deny anthropocosmic solidarity and openness.” (Hottois, 1996a, p. 108).

Here we turn back to bioethics, since it is the field of bioethics that institutionally embody this philosophical guidance. “It is in this new space that the guidance is increasingly institutionalized in the form of commissions, committees, declarations, laws and codes.” (Hottois, 1996a, p. 18) This institutional space allows for “procedural rules and institutions, with revisable conclusions, sensitive to empirical changes in content and context.” (Hottois, 2004, 29) In other words, techniques and symbols “can interact here without domination.” (Hottois, 2005, 29)^7^

It is within this bioethical institutionalization that Hottois also reconceptualizes the role of the philosopher of technoscience. This has to do with the fact that bioethical debates start from two basic assumptions. First, there is what I will call *technical pluralism*: bioethics has to recognize the temporality of technoscience and thus the open and creative process of technical change. Second, there is *symbolic pluralism*: the recognition that there is a plurality of symbolic systems in society, and that none can claim to be the final one. Both pluralisms imply specific functions for the philosopher.

First, Hottois sees the philosopher as tasked with defining bioethical concepts such as life or death, but these symbolic interventions should be judged in light of the practical operativity of language: “definitions and descriptions are immediately charged with a normative function, allowing or forbidding certain things to be done: admitting or rejecting certain research and certain technical applications.” (Hottois, 1999, p. 23) Thus, bioethical language does not reflect reality, but intervenes in reality by steering the technical pluralism of technoscience in certain directions. “Bioethical discourse is fundamentally practical, and all theoretical choices must be seen from a pragmatic point of view, i.e. from the point of view of its consequences for action.” (Hottois, 1999, p. 23).

Second, philosophers must refrain from taking sides in the debate, but rather “ensure the ethics of discussion, which requires that the views of the various stakeholders can be expressed freely and without constraint, and that they are considered and debated.” (Hottois, 1997, p. 490) For Hottois, this requires recognition of the existing symbolic pluralism in society. Bioethical debates should not be seen as leading to one correct answer, but must be guided in such a way that all relevant voices are taken into account. It must therefore be conceptualized as a “symbolic laboratory for experimenting with moral convictions” (Hottois, 1999, p. 25). In this sense, there is one meta-ethical position through which the philosopher does intervene, namely that of moral pluralism: any position that claims to have the ultimate ethical truth at the expense of all others cannot have the last word.^8^

However, one can object that this project of guidance remains vague. It is one thing to say what we must avoid, it is another to say what we must do. There are, however, a number of hidden underlying values at work in Hottois's framework that make this middle road more specific. In line with French biological philosophy of technology, Hottois seems to favor processes of *diversification*: guidance is good if it creates genuine room for more and novel diversity. “My hypothesis is that the technoscientific dynamic of emancipation from any given symbolic and biophysical constraint is in itself *good*, insofar as it coincides with a process of liberation, diversification, enrichment in the sense of the blossoming of possibilities and virtualities.” (Hottois, 1999, p. 41) Hottois also formulates this through two minimal requirements of any bioethical deliberation: “1) not to cause (unnecessary? physical?) suffering, not to add to the natural evil of living beings; 2) never to destroy or irrevocably modify a typical cosmic crystallization; above all, never to destroy a form of life definitively and absolutely.” (Hottois, 1996a, p. 39).

These two principles can be translated into two requirements to ensure proper diversification, when read in relation to symbols and techniques. While the first concerns the guiding of symbols, the second concerns the guiding of techniques. At first sight, the first principle seems to be a form of utilitarianism, where the minimal requirement of ethics is to avoid pain. As is often the case, the harder question of what kind of suffering is acceptable in certain circumstances is left open (‘unnecessary?’). However, the main stake here seems to be the liberal principle that no additional, more substantial symbolic values can be seen as absolutely valid, especially if they cause more harm. No static symbol should be introduced that restricts our behavior beyond the call to avoid suffering.

The second principle, in turn, concerns the limits of the openness of the technical: technical change and novelty should have their place, but not at the expense of existing forms of existence, ‘cosmic crystallizations’. Hottois also often formulates this principle as follows: the aim of bioethics is not to preserve specific ethical principles, but to preserve the capacity for ethics. This capacity is constantly threatened by technoscience, since its anti-theoretical principle (‘everything is possible’) is always also an “anethical” principle, since it risks destabilizing not only “a specific type of ethics (moral system), a specific value; it questions ethics as such, *ethicicity*: the status, legitimacy and value of ethics” (Hottois, 1988, p. 11). Specifically, it concerns bioethical questions where the human form is at stake, such as genetic modification. Here Hottois suggests as a guiding principle: “*Only those techno-scientific possibilities should be promoted that do not run the risk of seriously and irreversibly altering or even eliminating humanity’s ethical capacity*.” (Hottois, 1984a, p. 171).

For Hottois, this implies a limit to any kind of radical transhumanism: “Man is irreplaceable for two closely related reasons: man has value in himself and (or rather: because) man is the source of all value. It is through man, and through man alone, that what we call duty, morality and ethical capacity exist in the universe.” (Hottois, 1984b, p. 57) In this sense, there is a clear question that guides bioethical concerns: “does this or that technoscientific possibility risk diminishing, or even eliminating, the ethical capacity of the individual and of humanity?” (Hottois, 1984b, p. 59) For example, when we consider whether we are allowed to modify the body in such a way that it does no longer feels pain. For Hottois, this is out of the question because the capacity to feel pain is necessary for ethicicity.

One might object that this is a rather strong claim, and one that Hottois is prepared to make: “Outside of natural-cultural humanity there is no ethics: humanity and ethics are interdependent.” (Hottois, 1984a, p. 168) Is this not itself a symbolization that Hottois smuggles in here as an absolute? Hottois seems to fall back on a minimal, but fundamental conception of the human. Ethicicity requires the human form, and in particular what he calls *affectivity* or *love*. The former comes out of “a transcendental question about ethics: what are the conditions of possibility and validity of ethics as such? I believe that this questioning could rediscover affectivity as (at least) one of the ‘transcendentals’ of ethics.” (Hottois, 1988, p. 12) However, Hottois is not so forthcoming about what affectivity entails.

He says a little more about the latter, the concept of love, which he presents as a similar condition of possibility of ethicicity: “Man’s ethical choice proceeds from a vital source whose nature is not logotheoretical: *love*.” (Hottois, 1984a, p. 176) In this brief quotation, we already find a start to answer the above objection: ethicicity is grounded in love, but love is not to be understood as a symbol, but itself a biological process in the world. Therefore, there is no further justification to be given, since that would fall back on symbols. “If the source of humanity’s ethical choice is love, this choice no longer needs any other legitimation. *Love does not wait for a foundation*. It is self-sufficient; it is its own complete justification.” (Hottois, 1984a, p. 177) Hottois even sees it as “the only force that can counterbalance the temptations and powers of technique”, since it can offer an independent creative force through which symbols can be created: “Love must permeate the symbol, inspire discourse and writing, guide the symbolic order, and in this way - in a certain ideological way - influence the growth of technology.” (Hottois, 1984a, p. 176) Or to quote him more fully:

There is [besides the symbolic…] another source - not symbolic, and therefore not conceptual, not reasoned - of attachment to man. This source is not merely formal; on the contrary, it is dense and alive: *love*. Love is, in its own way, operative, as effective and active as technology. […] It is ultimately the experience of affectivity (which is neither of the order of the symbol - or at least not reduced to it - nor of the technical realm - that keeps us positively on the slope of technicist ideologization). Love, and by way of a non-logical consequence: ethics, is the only source of a certain light, very particular but very human (perhaps the most human), when the beacons of symbol and discourse have gone out. (Hottois, 1984a, p. 187)

He gives the following bioethical example: how should we treat human bodies after they die? The purely technical response sees bodies as mere reservoirs of organs to be harvested. Love, on the other hand, revolts against such an instrumental view, and sees bodies – affectively – as more than mere objects, as objects that demand our respect.

Although at first sight this preservation of ethicicity seems very anthropocentric, aimed to preserve human beings as they exist now, this preservation also extends to future generations: we must preserve not only our own capacity for ethics, but also that of future generations. We should give them the freedom to decide their own destiny:

Our concern for the future must not bind future generations to a world less rich in opportunity, and therefore in freedom, than our own. Our concern for the future must not lead us to close off forever avenues that we have not chosen for our own reasons and feelings. This is why a concern for the future implies, once again, a concern for the past, the transmission of the legacy of the possibilities of the past. (Hottois, 1996a, p. 110)

This quotation shows how this preservation of ethicicity also implies a wider preservation of diversification. For instance, it extends to technologies: “If we are not careful, the best technology for one environment may be the worst for another. It is therefore important to preserve technological diversity alongside biological and cultural diversity. If we care about the future, we must also care about the past.” (Hottois, 1996a, p. 109).

Finally, it extends to ecological diversity: the preservation of “cosmic crystallizations”, as quoted earlier. Here Hottois makes perhaps his most daring proposal. At first sight, there is a tension between this plea to preserve all forms and his conceptualization of cosmic time. Cosmic time is a constant process of living forms that appear and disappear again. Is the attempt to preserve them not an attempt to halt this change? Aware of this tension, Hottois suggests that the preservation of ecological diversity should take the form of the preservation of *accessible memory to the past*:

How can we combine technoscience and all that it entails with love for others? How can we combine active curiosity about others with awe and respect for them? How can we produce all that is possible without destroying what is already possible, in order to create an ever richer world? Perhaps the solution lies in a kind of inversion: saving the past without blocking future possibilities, preserving it as an accessible memory, not acting as if the only form of survival were the perpetuation of an identity that is always present, monolithic, immobile and time-negative. (Hottois, 1996a, p. 21)

The past and the diversity of forms are to be preserved, not as actually existing entities, but as accessible possibilities that exist ‘virtually’. This is where technosciences themselves can play a role. Not only are they valuable to create novel forms, but they also have been “developing powerful capacities to remember, to record, and even to reanimate the lost and silent traces of the past.” (Hottois, 1999, p. 179) This preservation of forms, moreover, extends to cultural practices. Although they may not survive in actuality, we must preserve them as accessible memory: “Just as we do not know whether the exploitation of a certain wild plant species that is no longer of interest will one day give rise to a promising possibility, so we cannot know whether in the future man will not find a new stimulus or a salutary lesson in the contemplation of a certain tradition that is now obsolete.” (Hottois, 1996a, p. 108) To make this more concrete, we can think of practices such as seed banks to save plants from extinction or technical recordings to preserve dying cultural practices.

Here, too, Hottois seems to endorse a set of hidden premises concerning why we should value (the preservation of) these diverse virtual life forms. More generally, this question opens up a prominent debate in bioethics and environmental philosophy about the ultimate grounds for our valuing of nature (O’Neill, Holland and Light 2008). Is nature valuable because it has value for humans (anthropocentrism) or does it have value in its own right (ecocentrism)? Insofar as Hottois takes an explicit position in this debate, it is not to be found in this classical opposition. Rather, Hottois’s starting point is an “anthropocosmic solidarity”, which “means that man is not essentially alien to the cosmos that surrounds him; on the contrary, as a natural species, he is a product of that cosmos” (Hottois, 1990b, p. 165). This idea has ethical ramifications for Hottois, in particular that nature is valuable because man is valuable: “If man has value, and if he is an integral part of a natural evolution and environment, these cannot be completely beyond value, beyond dignity.” (Hottois, 1990b, p. 166).

Hottois’s main inspiration for this position is Hans Jonas’ *The Imperative of Responsibility* (1984; see Hottois, 2002b). Echoing many of Hottois’s themes, Jonas argues that traditional ethics fails to address the long-term, planetary-scale risks of modern technology. As an alternative, Jonas proposes a ‘principle of responsibility’, a precautionary ethics that prioritizes the long-term survival of life. While broadly anthropocentric - grounding responsibility in human survival - Jonas’ focus on the ecological vulnerability of life simultaneously aligns with ecocentric concerns. Jonas’ philosophy thus offers Hottois a precautionary ethics that can serve as a pragmatic bridge between anthropocentrism and ecocentrism, extending moral responsibility beyond immediate human interests to the long-term viability of the biosphere.

But Hottois immediately adds another theme of his own, namely the dynamic nature of all ethical principles. To decide whether Hottois is an anthropocentrist or an ecocentrist is in a sense impossible, because he does not believe in fixed norms. So, while he may have an ecocentric position today, this could change tomorrow. Ethical norms should not be seen as eternally fixed, but rather as “fundamentally pragmatic in the sense that they solve […] problems of pluralistic social life provisionally; they are always revisable […]. They constitute an evolving and open ethics” (Hottois, 1990b, p. 193).

## Conclusion

This article has attempted to give an overview of the philosophy of Gilbert Hottois. It starts from a metaphilosophy of language, that diagnoses twentieth-century philosophy as fixated on language at the expense of technology. As an alternative, Hottois developed a philosophy of technoscience, which reinterprets science as primarily an intervening and technical activity rather than a contemplative and theoretical one. Hottois sought to articulate the nature of this technicity through a philosophy of time, reflecting on the specific temporality of technoscience as distinct from human history. This temporality of technoscience provoked the need for ethical reflection, since technoscience is constantly changing and transforming the world. This led to Hottois’s engagement with bioethics, in which he sought to develop a framework capable of ‘guiding’ technoscience. Aiming to avoid both complete symbolic closure and complete technical openness, this guidance is concerned with the preservation of diversity, especially the human capacity for ethics, ethicicity.

To conclude, we can return to the multiple legacies of Hottois’s work. Hottois’s concept of technoscience has been taken up by several authors (Lyotard, 1986; Latour, 1987; Stiegler, 1994; Haraway, 1997; Ihde, 2009; Bensaude-Vincent 2009), and his work can be read as representative of the practice turn in philosophy of science (Soler et al., 2014; Potters & Simons, 2023). What this article makes clear, however, is that his practice turn also implies, first and foremost, a move from a philosophy of science that poses epistemological questions to one that poses ethical and political questions (see Simons 2025). In this sense, Hottois’s real influence may lie more in his hidden footprints in Dutch technology ethics, in particular through the work of two authors strongly associated with the empirical turn in the philosophy of technology: Achterhuis (2001) and Peter-Paul Verbeek (2005). For example, much of Achterhuis’s work in the 1990s drew heavily on Hottois’s work, especially his reflections on the distinction between symbols and technology (Achterhuis, 1998, pp.235–254), as well as Hottois’s ambition to offer a way out by ‘guiding’ technology through ethics (Achterhuis, 1996, pp.39–40).

In a later text presenting his program of the empirical turn, Achterhuis argues that “with Hottois we want to hold on to the special place that the technosciences occupy in technological culture. The technosciences are about more than language games, symbolic exchange, or social power struggles.” In order to recognize the specificity of the non-symbolic side of technoscience, philosophy must abandon its ambition to develop an overarching and a priori ethical theory of technology and instead develop a practice that is “best described by the concept of ‘accompagnement’ (‘guidance’) borrowed from Hottois, which clearly contrasts with the ‘leading of technology’ that a classical philosopher of technology like [Karl] Jaspers still advocates” (Achterhuis, 2004, p. 28).

Peter-Paul Verbeek, who was a student of Achterhuis, picks up on this message and borrows Hottois’s concept of ‘guidance’ (*accompagnement*), to argue for a shift in technology ethics, moving away from “an external standpoint vis-à-vis technology, aiming only to either reject or accept a new technology” towards an “ethics of technology” that “aims to accompany technological developments” (Verbeek, 2011, p. 95). He reiterates this claim in his recent ‘Guidance Ethics Approach’ (Verbeek and Tijnk 2020), where he similarly asserts that the value of technology ethics lies not only “in the approval or rejection of technologies, but also in the guidance of their development, implementation, and use”. Hence, “the focus is rather on ‘accompaniment’.” (Verbeek 2021, p.48).

In this Dutch tradition, however, Hottois’s analysis of the relationship between technoscience and temporality has been taken up only selectively. In particular, the Dutch have emphasized the idea that the ethical evaluation of technology is not a static but a dynamic activity: not only is one shooting *at* a moving target (technology changes), but one is also shooting *from* a moving platform (morality changes). We see this idea at work in frameworks that build on mediation theory, such as “technomoral change” (Swierstra et al., 2009) and “value dynamism” (Kudina, 2019), which aim to map how technology and morality change and influence each other over time. This is indeed a message that Hottois would welcome.

What is lost in translation, however, is the insight that not only do technologies and symbols always change dynamically over time, but that their temporalities are also different. In other words, a proper and critical analysis of technologies requires the recognition and empirical study of the different temporalities of technologies and symbols. Central to Hottois’s concern is precisely the fact that there is always the potential for a mismatch between these temporalities: either by symbolic systems being too closed and slow, or by technical operations being too open and fast, with the risk of biophysically alienation. This selective adoption may be related to the ambiguity of the ‘practice turn’ described earlier, which can be understood as either a methodological or an ontological claim, i.e. either the claim that science is a practical activity or the claim that we should study science by studying scientific practices. And while Hottois’s idea of guidance is deeply compatible with the methodological version, the opposition between symbol and technique, and all that follows from this opposition, is of a much more speculative nature.

In this sense, inspired by Hottois, there is room for a complementary philosophy of technology that focuses on a critical analysis of the different temporalities involved in technologies and how they (mis)align with cultural and symbolic temporalities. Inspired by Bachelard (1936) and Henri Lefevre (1992), we could speak of a *technological rhythmanalysis*, i.e. an analysis of how the different temporal rhythms of technologies and humans mediate each other, and how a mismatch can lead to ethical, social and political concerns. In other words, we find here a potentially different normative criterion for evaluating technologies: do the temporalities of an existing or proposed technology match the existing or desired temporalities of human existence? Such a framework could recognize the structural difference between technology and culture, while acknowledging their dynamic nature. Although Hottois’ work can be a starting point for this effort, such a technological rhythmanalysis should be more broadly grounded in the French tradition of biological philosophy of technology, whose work can be read as a toolbox for such a novel project.^9^

## Data Availability

Not applicable.

## Footnotes

1 Quotations from Hottois’ works are translated by the author.

2 For the classical formulation of the claim about the primacy of technology, see Forman (2007).

3 For a contemporary articulation and development of this point, see Bensaude-Vincent et al. (2011, 2017).

4 In his textbook, Hottois also identifies a number of more recent developments that further complicates contemporary technoscience. In addition to the increasing complexity of science and technology, as well as an explosion of societal concern and philosophical reflection, he points to how “the *informational* and *molecular-biological* revolutions have transformed the relationship between man and technology. These revolutions produce ‘machines’ and an ‘artificial environment’ in a completely new sense: the living itself is ‘machined’ (by genetic engineering) and the artificial environment becomes immaterial (computer networks, cyberspace, virtual reality, etc.).” (Hottois, 1997, p. 495).

5 As I argued elsewhere (see Simons 2022a), there are strong analogies with Lyotard’s philosophy of technoscience. In *La condition postmoderne* (1979) Lyotard claims that postmodernity inaugurates the end of the belief in metanarratives: scientific research is no longer driven by historical narratives but by ‘performativity’. This logic of performativity echoes Hottois’s notion of operationality. In later works, Lyotard will take up the concept of technoscience, after he “read a young Belgian philosopher of language who complained that Continental thought […] had replaced the paradigm of referentiality with one of adlinguisticity” (Lyotard, 1986, p. 2). Similar to Hottois, Lyotard asserts how: “We are like Gullivers in the world of technoscience: sometimes too big, sometimes too small, but never the right size.” (Lyotard, 1986, p. 79) And in dramatic form, Lyotard will use a “postmodern fable” (Lyotard, 1993) to sketch cosmic time from the Big Bang to the history of humanity, with the suggestion that human history will end, at least, with the explosion of the sun. “Thus the last exodus of the negentropic system was prepared far from the Earth. What the Human and his Brain could look like, or rather the Brain and his Human, when they left the planet forever, that history did not say” (Lyotard, 1993, p. 86).

6 Here Hottois’s philosophy of technoscience diverges from the ‘practice turn’ in philosophy of science, which typically ignores ethical concerns. Hacking, for instance, justifies not engaging with ethics on the grounds of ignorance: “qua philosophers of science, they are no better qualified to discuss such matters than is a truck driver or a Xerox repair person” (Hacking, 1999, p. 169). Hottois is not convinced: “Of course it is inappropriate to ask someone to speak about what they do not know. But we can ask them if they have the right to remain ignorant about it.” (Hottois, 2005, 86) Instead, Hottois’s philosophy of technoscience here comes close to the ethical frameworks developed in philosophy of technology (Verbeek, 2008, 2011).

7 In addition to bioethics, Hottois also saw human rights as an institutionalization of this guidance. According to him, “the philosophy of human rights, especially the Universal Declaration of Human Rights (UDHR) of 1948, is the best expression so far of what is needed in terms of symbolic support for the technosciences” (Hottois, 1996a, p. 217). In contrast to the original Declaration of 1789, the Declaration of 1948 is important because “the former still seems to be based on transcendent references” while “a study of the discussions that led to the text of the new Declaration in 1948 shows that the authors of the text (representatives of the ‘free world’) voluntarily and consciously renounced this kind of founding reference” (Hottois, 1999, p. 113). Human rights are thus flexible enough to guide technoscience if they are read as open signifiers to which different parties appeal in different circumstances. “The Declaration of Human Rights thus simply takes advantage of *a fundamental property of language* […] which consists in the possibility of using, to varying degrees, *a single signifier for a variable and shifting number of signifieds*.” (Hottois, 1996a, p. 44).

8 On this point, Hottois seems to have been inspired by the ‘discourse ethics’ of Karl-Otto Apel (1988) and Jürgen Habermas (1990), which runs as an underlying theme through Hottois’s work.

9 Of course, we need not confine ourselves to Bergson, Simondon or Stiegler. In addition to the work of Bachelard and Lefevre already mentioned, one could also refer to other work as well, such as the ‘dronology’ of Virilio (1977), the work on ‘resonance’ of Rosa (2010) or the work of Bernadette Bensaude-Vincent on ‘timescapes’ (Bensaude-Vincent, 2021, 2022).

## References

[CR1] Achterhuis, H. (1996). Filosofie van de technologische cultuur. Onderzoeksprogramma 1996–2000. *Filosofie*, *6*(5), 36–40.

[CR2] Achterhuis, H. (1998). *De erfenis van de utopie.* Ambo.

[CR4] Achterhuis, H. (Ed.). (2001). *American philosophy of technology: The empirical turn*. Indiana University Press.

[CR3] Achterhuis, H. (2004). De transformerende kracht van de technologie. *Filosofie*, *14*(2), 27–30.

[CR5] Allen, B. (2023). *Living in time: The philosophy of Henri Bergson*. Oxford University Press.

[CR6] Apel, K. (1988). *Diskurs und Verantwortung: Das Problem des Übergangs Zur postkonventionellen m*. Suhrkamp.

[CR7] Bachelard, G. (1934). *Le nouvel esprit scientfiique.* Alcan.

[CR8] Bachelard, G. (1936). *La dialectique de la durée*. Boivin.

[CR9] Bardin, A. (2015). *Epistemology and political philosophy in Gilbert Simondon: Individuation, technics, social systems*. Springer.

[CR10] Benhabib, S. (1986). *Critique, norm, and utopia: A study of the foundations of critical theory*. Columbia University.

[CR88] Bensaude-Vincent, B. (2009). *Les vertiges de la technoscience: Façonner le monde atome par atome*. Éditions La Découverte.

[CR11] Bensaude-Vincent. (2021). *Temps-paysage*. Le Pommier.

[CR12] Bensaude-Vincent (2022). Rethinking time in response to the anthropocene: From timescales to timescapes. *The Anthropocene Review*, *9*(2), 206–219.

[CR13] Bensaude-Vincent, B., Loeve, S., Nordmann, A., & Schwarz, A. (2011). Matters of interest: The objects of research in science and technoscience. *Journal for General Philosophy of Science*, *42*(2), 365–383.

[CR14] Bensaude-Vincent, B., Loeve, S., Nordmann, A., & Schwarz, A. (2017). *Research objects in their technological setting*. Routledge.

[CR15] Bergson, H. (1932). *Les Deux Sources de la morale et de la religion.* Alcan.

[CR16] Chabot, P. (2003). *La philosophie de Simondon*. Vrin.

[CR17] Clarizio, E. (2021). *La Vie technique. Une philosophie biologique de La technique*. Hermann.

[CR18] Clarizio, E. (2023). Technical invention as biological function. *Techné: Research in Philosophy and Technology*, *27*(1), 21–41.

[CR19] De Dijn, H. (2000). De donkere transcendentie Van Prometheus. Tijdschrift Voor Filosofie, 62(4), 743–751

[CR20] Demeester, W. (Ed.). (1987). *Bioéthique dans les années ‘90. Tome 1: Compte rendu des groupes de travail*. Omega Editions.

[CR21] Dumoncel, J. C. (1985). L’essence double du langage Selon Gilbert Hottois. *Revue Philosophique De Louvain*, *83*(2), 262–266.

[CR22] Feenberg, A. (2018). *Technosystem: The social life of reason*. Harvard University Press.

[CR23] Feyles, M. M. (2022). Two problems of the biological philosophy of technology. *Philosophy & Technology*, *35*(2).

[CR24] Forman, P. (2007). The primacy of science in modernity, of technology in postmodernity, and of ideology in the history of technology. *History and Technology*, *23*(1/2), 1–152.

[CR25] Goffi, J. (1988). Gilbert Hottois, Penseur de La technique. *Laval Théologique Et Philosophique*, *44*, 3, 327–337.

[CR26] Habermas, J. (1987). *The theory of communicative action: Volume 2, lifeworld and system: A critique of functionalist reason*. Beacon Press.

[CR27] Habermas, J. (1990). *Moral consciousness and communicative action*. Polity.

[CR28] Hacking, I. (1983). *Representing and intervening*. Cambridge University Press.

[CR29] Hacking, I. (1999). *The social construction of what?* Harvard University Press.

[CR30] Haraway, D. (1997). *Modest_Witness@Second_Millennium.FemaleMan_Meets_OncoMouse: Feminism and technoscience*. Routledge.

[CR89] Henry, M. (1987). *La barbarie*. Grasset

[CR31] Hottois, G. (1976). *La philosophie du langage de Ludwig Wittgenstein*. Editions de l’Université de Bruxelles.

[CR32] Hottois, G. (1979). *L’inflation du Langage Dans La philosophie contemporaine: Causes, formes et limites*. Ed. de l’Université de Bruxelles.

[CR33] Hottois, G. (1980). Secondarity: A central concept of contemporary philosophy. *Metaphilosophy*, *11*(2), 134–137.

[CR34] Hottois, G. (1981). *Pour une métaphilosophie du langage*. Vrin.

[CR35] Hottois, G. (1984a). *Le Signe et La technique: La philosophie à L’épreuve de La technique*. Aubier.

[CR36] Hottois, G. (1984b). *Pour une éthique dans un univers technicien*. Ed. de l’Université de Bruxelles.

[CR49] Hottois, G. (Ed.). (1988). *Evaluer La technique: Aspects éthiques de La philosophie de La technique*. Vrin.

[CR37] Hottois, G. (1990a). Die natur der sprache: Von der ontologie Zur technologie. *Studia Leibnitiana*, *22*(2), 184–193.

[CR38] Hottois, G. (1990b). *Le paradigme bioéthique.* De Boeck.

[CR39] Hottois, G. (1993). *Simondon et La philosophie de La culture technique*. De Boeck.

[CR40] Hottois, G. (1996a). *Entre Symboles et technosciences: Un itinéraire philosophique*. Champ Vallon.

[CR41] Hottois, G. (1996b). Articuler le temps entre phénoménologie et technologie, 173–188. In: Weyembergh, M., & Hottois, G. (Éds.). (1996). *Temps cosmique, histoire humaine*. Vrin.

[CR42] Hottois, G. (1997). *De La renaissance à La postmodernité: Une histoire de La philosophie moderne et contemporaine*. De Boeck.

[CR43] Hottois, G. (1999). *Essais de philosophie bioéthique et biopolitique*. Vrin.

[CR44] Hottois, G. (2002a). *Species Technica*. Vrin.

[CR86] Hottois, G. (2004). *Qu’est-ce que la bioéthique?* Vrin.

[CR51] Hottois, G. (Ed.). (2002b). *Hans Jonas: Nature et responsabilité*. Vrin.

[CR87] Hottois, G. (2005). La science entre valeurs modernes et postmodernité: conférence au Collège de France. Vrin.

[CR46] Hottois, G. (2017). *Philosophie et idéologies trans/posthumanistes*. Vrin.

[CR48] Hottois, G. (2022). *La science-fiction. Une introduction historique et philosophique*. Vrin.

[CR50] Hottois, G. (Ed.). (2000). *Philosophie et science-fiction*. Vrin.

[CR52] Hottois, G., & Missa, J. (Eds.). (2001). *Nouvelle encyclopédie de bioéthique: Médecine, environnement, biotechnologie*. De Boeck.

[CR53] Hottois, G., & Parizeau, M. (Eds.). (1993). *Les Mots de La bioéthique: Un vocabulaire encyclopédique*. De Boeck.

[CR54] Ihde, D. (2009). *Postphenomenology and technoscience: The Peking university lectures*. State University of New York.

[CR55] Jonas, H. (1984). *The imperative of responsibility*. University of Chicago Press.

[CR56] Kudina, O. (2019). *The technological mediation of morality: Value dynamism and the complex interaction between ethics and technology*. University of Twente.

[CR57] Latour, B. (1987). *Science in action: How to follow scientists and engineers through society*. Harvard University Press.

[CR58] Lefebvre, H. (1992). *Éléments de rythmanalyse: Introduction à La connaissance des rythmes*. Syllepse.

[CR59] Loeve, S., Guchet, X., & Bensaude-Vincent, B. (Eds.). (2018). *French philosophy of technology: Classical readings and contemporary approaches*. Springer.

[CR60] Lyotard, J. (1979). *La condition postmoderne: Rapport Sur Le savoir*. Ed. de Minuit.

[CR61] Lyotard, J. (1986). *Le postmoderne expliqué aux enfants: Correspondance 1982–1985*. Galilée.

[CR62] Lyotard, J. (1993). *Moralités postmodernes*. Galilée.

[CR63] Missa, J. (2022). Préface: Gilbert Hottois et la science-fiction, 7–46. In: Hottois (2022).

[CR64] Nordmann, A. (2007). If and then: A critique of speculative ethics. *NanoEthics*, *3*(1), 31–46.

[CR65] O’Neill, J., Holland, A., & Light, A. (2008). *Environmental values*. Routledge.

[CR66] Potters, J., & Simons, M. (2023). We have never been new experimentalists: On the rise and fall of the turn to experimentation in the 1980s. *HOPOS*, *13*(1), 91–119.

[CR67] Rorty, R. (Ed.). (1970). *The linguistic turn: Recent essays in philosophical method*. University of Chicago Press.

[CR68] Rosa, H. (2010). *Alienation and acceleration. Towards a critical theory of Late-Modern Temporality*. NSU.

[CR69] Rouse, J. (1987). *Knowledge and power: Toward a political philosophy of science*. Cornell University Press.

[CR70] Schecter, D. (2010). *The critique of instrumental reason from Weber to habermas*. Continuum.

[CR71] Schlanger, N. (2023). *L’invention de la technologie. Une histoire intellectuelle avec André Leroi-Gourhan*. PUF.

[CR72] Sebbah, F. (2010). *Qu’est-ce Que La technoscience?* Les Belles Lettres.

[CR73] Simondon, G. (1958). *Du mode d’existence des objets techniques*. Aubier Montaigne.

[CR74] Simons, M. (2022a). Jean-François lyotard and postmodern technoscience. *Philosophy & Technology*, *35*(2).

[CR75] Simons, M. (2022b). Playing god: Symbolic arguments against technology. *NanoEthics*, *16*(2), 151–165.

[CR90] Simons, M. (2025 - forthcoming). Political Philosophy of Experimentation. *European Journal for Philosophy of Science*10.1007/s13194-025-00665-1 PMC12174169 40538681

[CR76] Soler, L., Zwart, S., Lynch, M., & Israel-Jost, V. (Eds.). (2014). *Science after the practice turn in the philosophy, history, and social studies of science*. Routledge.

[CR77] Stiegler, B. (1994). *La technique et Le temps. 1: La faute D’Épiméthée*. Galilée.

[CR78] Swierstra, T., Stemerding, D., & Boenink, M. (2009a). Exploring Techno-moral Change: The Case of the ObesityPill, 119–138. In: *Evaluating New Technologies*, edited by P. Sollie & M. Duwell. Springer.

[CR79] Swierstra, T., Van Est, R., & Boenink, M. (2009b). Taking care of the symbolic order. How converging technologies challenge our concepts. *NanoEthics*, *3*(3), 269–280. 20234840 10.1007/s11569-009-0080-0 PMC2837246

[CR80] Verbeek, P. (2005). *What things do: Philosophical reflections on technology, agency, and design*. Pennsylvania State University Press.

[CR81] Verbeek, P. (2008). Obstetric ultrasound and the technological mediation of morality: A postphenomenological analysis. *Human Studies*, *31*(1), 11–26.

[CR82] Verbeek, P. (2011). *Moralizing technology: Understanding and designing the morality of things*. University of Chicago Press.

[CR83] Verbeek, P. (2020). The Empirical Turn, 35–54. In: Shannon Vallor (Ed.) *The Oxford Handbook of Philosophy of Technology*. Oxford University Press.

[CR84] Verbeek, P., & Tijink, D. (2020). *Guidance Ethics Approach.* ECP.

[CR85] Virilio, P. (1977). *Vitesse et Politique: essai de dromologie*. Galilée.

